# Validity and reproducibility of a tool for assessing clinical competencies in physical therapy students

**DOI:** 10.1186/s12909-018-1377-x

**Published:** 2018-11-23

**Authors:** Martha-Rocío Torres-Narváez, Olga-Cecilia Vargas-Pinilla, Eliana-Isabel Rodríguez-Grande

**Affiliations:** 1 Universidad del Rosario, Bogotá, Colombia; 2 Escuela de Medicina y Ciencias de la Salud, GI Ciencias de la Rehabilitación, Bogotá, Colombia; 3 Programa de Fisioterapia, Bogotá, Colombia

**Keywords:** Clinical competencies, Physical therapy, Student performance, Clinical education, Professionalism

## Abstract

**Background:**

The evaluation of competencies in the clinical field is essential for health professionals, as it allows the acquisition of these competencies to be tracked. The objective of this study was to create and evaluate the validity and reliability of a tool for measuring clinical competencies in physical therapy (PT) students to assess the quality of their performance in a professional context.

**Methods:**

A descriptive study was designed. The Measurement Tool for Clinical Competencies in PT (MTCCP) was developed based on the evaluation of 39 experts: 15 clinicians and 24 instructors. The content validity *was* evaluated using the Content Validity Index (CVI). Three professors were invited to apply the tool to 10 students. Cronbach’s alpha, exploratory factor analysis, and the intraclass correlation coefficient were used to determine the reliability and validity of the scale.

**Results:**

The CVI was positive—higher than 0.8. Principal component analysis confirmed the construct validity of the tool for two main factors: clinical reasoning (first factor) and professional behavior (second factor). With regard to reliability, the MTCCP achieved an internal congruence of 0.982. The inter-evaluator reproducibility for clinical reasoning, professional behavior, and the total MTCCP score was almost perfect; the ICCs were 0.984, 0.930, and 0.983, respectively.

**Conclusions:**

The MTCCP is a valid and reliable instrument for assessing the performance of PT students in hospital settings and can be used to determine what skills students feel less confident using and what additional training/learning opportunities could be provided. Further research is needed to determine whether the MTCCP has similar validity and reproducibility in other Spanish-speaking national and international PT programs.

**Electronic supplementary material:**

The online version of this article (10.1186/s12909-018-1377-x) contains supplementary material, which is available to authorized users.

## Background

The goal of university education is to expand, broaden, and transform the mind and to prepare students to effectively address problems [1]. Higher education should be centered on students and should contribute to their personal growth and to their intellectual, psychological, and moral development [2]. For the health professions, the Institute of Medicine has proposed a set of core competencies for all health disciplines that will allow clinicians to deliver patient-centered care as members of an interdisciplinary team; these competencies emphasize evidence-based practice, quality improvement approaches, and informatics [3].

Competency-based education has thus been proposed as a means to optimize the preparation of health professionals [4]. The competency-based approach is focused on outcomes related to the skills, knowledge, and attitudes of graduates [5] that will allow them to work as competent professionals at the national or international level [6, 7]. Competence-based learning is based on the capacity and responsibility of each student and on the development of the student’s autonomy [2]; it requires specific learning methodologies, monitoring, and tutoring as well as competency-based assessment methods [8].

At the entry level, educational programs in physical therapy (PT) integrate theory, evidence, and practice with the aim of producing knowledgeable, helpful, confident, adaptable, and reflective professionals who can practice independently and autonomously to meet their patients’ or clients’ needs, as supported by evidence [9, 10]. To achieve this end, the curriculum must ensure that graduates will be able to demonstrate the established entry-level (undergraduate) competencies based on the priorities of the educational program, institution, and country while conforming to the national and international standards of the PT profession [4].

Due to the competencies’ focus on performance, the formal assessment of students should be modified according to the practice setting. The evaluation of competencies in clinical areas is essential for PT: it allows the acquisition of competencies to be supervised, thus helping to improve competency levels and practice standards for new graduates [11]. The evaluation of clinical competencies takes into account the performance of professionals during patient or client interaction with respect to the clinical reasoning that is applied to decision making. This evaluation includes the conceptual commands, expert judgment, team work, communication, motor skills, and professionalism that are necessary for providing health services [11–13].

The current research on educational evaluation is oriented toward the way knowledge acquisition is integrated with strategies for the measurement and quantification of capacities in technology and science. To comprehensively evaluate students in a specific time and context, the process of evaluating clinical competencies needs to be elevated to a more formal and complex level [14–17].

Current evaluation tools follow the recommendations of the World Confederation for Physical Therapy (WCPT) and the American Physical Therapy Association (APTA) for assessing the competencies of physical therapists at the undergraduate level (commonly referred to as the entry level) [18, 19]. These tools group competencies into professional training, patient management, and resource scheduling and management. The grading of the performance level is carried out using numerical, ordinal, or interval scales; a PT student must reach a pre-determined performance level to graduate from an undergraduate program [8, 20–23].

We conducted a review of the literature and found that no studies have examined the tools for measuring clinical competence in a Spanish-speaking context. In addition, the tools that have been created for an English-speaking context have not been validated in Spanish. Therefore, there is a need to create and evaluate a tool that assesses student competencies and the quality of student performance in a professional Spanish-speaking context. Based on the above discussion, this study seeks to determine the validity and reliability of the Measurement Tool for Clinical Competencies in PT (MTCCP).

## Methods

### Development of the MTCCP

This was a descriptive study of the validity and reproducibility of the MTCCP. To establish the theoretical basis for the development of the MTCCP, we reviewed and analyzed technical documents produced by the WCPT, APTA, and the Commission on Accreditation in Physical Therapy Education (CAPTE). These documents describe the performance evaluation of PT students and tools such as the Clinical Performance Instrument (CPI) and the Clinical Internship Evaluation Tool (CIET). We also considered the professional competencies established by the ASCOFI. This document analysis allowed us to identify conceptual references for competencies, constructs, criteria, items, and evidence that could guide our design of the MTCCP.

The MTCCP assesses the knowledge, attitudes, skills, and abilities of PT students at clinical practice sites during decision making, i.e., clinical competency. Within this process, the clinical instructor (CI) plays the fundamental role of the judge who verifies the students’ learning achievements. Therefore, instructors must have the conceptual and methodological ability to evaluate the acquisition of clinical competencies [24].

The MTCCP defines two assessment dimensions: professional behavior and clinical reasoning. Each dimension has 10 items (Additional file 1: Appendix). We defined professional behavior as a set of attitudes and behaviors reflecting a physical therapist’s ethical commitment in providing health services. Professional behavior involves the consistent demonstration of values, including altruism, excellence, care, ethics, respect, communication, and accountability, related to professional performance [8].

Clinical reasoning refers to the critical thinking process that physical therapists engage in when making decisions. This reasoning is reflected in a set of cognitive and psychomotor skills used in decision making, including examination, evaluation, diagnosis, prognosis, and intervention [25–27]. Clinical reasoning includes a student’s skills in gathering information from patients, including their medical history, and in conducting physical examinations. In addition, clinical reasoning involves the following: the ability to prepare clinical reports based on knowledge and understanding of pathology; the interpretation of complementary clinical tests; and the assessment of the impact that a particular condition has on movement and functioning capabilities [28]. Furthermore, clinical reasoning includes the ability to make clinical judgments and solve problems and to combine various elements to provide a diagnosis and design a treatment plan.

In its structure, the MTCCP follows the guidelines of an evidence-based model that establishes the methodology for determining the competencies to be evaluated and describes the aspects to be included in the evaluation: statements or items and evidence [29]. An item is defined as a general statement about the facts that students should master with regard to clinical reasoning and professional behavior. Evidence represents behaviors or observable products that allow the verification of students’ performance levels in relation to expected levels with regard to actions, conceptual background, and motor and cognitive abilities. Clinical instructors examine each piece of evidence and then assign a score to each item to obtain an overall grade.

The student evaluation conducted with the MTCCP is based on a competency-based analysis. This analysis is a dynamic longitudinal process that monitors a person’s use of the knowledge, skills, attitudes, and sound judgment relevant to the profession with the aim of becoming completely professionalized [30].

The MTCCP employs a discrete measurement scale from 1 to 5. The tool includes key aspects that determine students’ autonomy: the required monitoring level and the degree of fulfillment of their competencies or functions at a practicum site [29]. The CIs evaluate each item separately based on the evidence pertaining to it. The instructors score their students’ performance, taking into account each student’s level of compliance with the expected profile of a recent PT graduate in relation to the evidence proposed for each item and the degree of supervision required.

The maximum MTCCP score is 100 points, which equates to a grade of 5.0, the highest possible grade for a student. To determine a student’s performance grade, the CIs take the average score for the items in each dimension and multiply them by the factor corresponding to clinical reasoning and professional behavior; this figure is then added to the total for each dimension to obtain the final grade.

### Participants

To determine the measurement properties, three distinct convenience samples were used. To evaluate the content validity, we calculated the sample size for a minimum concordance of 0.80, a reliability of 95%, and a power of 99%; we established that a sample of 32–40 people would allow the tool’s content to be validated.

The evaluation of the content validity was carried out by a group of 39 experts who were classified as clinical (15) or academic (24) and selected from the ASCOFI and PT training programs in Colombia.

In the second stage of the content validation, we selected 11 experts from the group of 39 evaluators according to their affiliation, geographical location, and availability to travel to the consensus meeting.

Finally, to evaluate reliability, we estimated the sample size for three evaluators with a reliability of 95% and a power of 80%. We established that a sample of 10 students would allow inter-evaluator reproducibility. Three instructors (with over 10 years of clinical experience and over 5 years of teaching experience) assessed 10 students engaged in clinical practice in two tertiary and quaternary care institutions in the areas of hospitalization, intensive care, and outpatient visits.

### Psychometric properties

#### Content validity

This study was conducted between September 2014 and March 2016. Thirty-nine experts agreed to participate and accordingly signed a confidentiality agreement. The evaluators received via e-mail the tool and conceptual framework to evaluate the relevance, sufficiency, coherence, and clarity of each item. Their evaluations were based on a Likert scale from 1 to 4, where 1 was the lowest grade for each item and 4 the highest. The evaluators were also able to suggest any other items they considered necessary. To improve clarity and prevent bias, we summarized these suggestions to determine whether our items should be revised or adjusted in terms of coherence, length, or redundancy.

In the second stage, 11 experts, using the Delphi technique [31], assessed the structure and content of each item, the evidence of the tool, and the guidelines. They used the following scale to evaluate each item: 2, essential; 1, useful but not essential; and 0, unnecessary. We calculated an agreement index with a cut-off point of 90%; items below the cut-off point were adjusted or eliminated by consensus. On applying this consensus approach, we determined that 60% of the final grade would correspond to the clinical reasoning dimension and 40% to the professional behavior dimension.

#### Reliability

We trained three instructors in the standardized implementation of the tool. The instructors evaluated 10 students at two different times, with a one-week interval between the evaluations. The instructors’ evaluations were not disclosed to the others when all three instructors were simultaneously evaluating student performance during the consultation process of a previously assigned patient. We observed the evaluations at each practicum site to verify the proper implementation of the evaluation protocol.

#### Statistical analysis

##### Content validity

We evaluated the relevance, sufficiency, pertinence, coherence, and clarity of each item using the content validity index (CVI). The CVI varies between + 1 and − 1, where higher positive scores indicate higher content validity.

##### Construct validity

We assessed the construct validity by means of exploratory factor analysis. Bartlett’s test of sphericity and the Kaiser-Meyer-Olkin (KMO) measure of sampling adequacy were used to confirm the appropriateness of the factor analysis. A KMO value > .8 is considered good, indicating the strength of the correlation between items. Next, Bartlett’s test of sphericity was conducted. Finally, principal component analysis using Varimax rotation was used as a dimension reduction technique [32].

##### Reliability

We assessed the MTCCP’s internal consistency using Cronbach’s alpha coefficient: α ≥ 0.9 was considered excellent; 0.8–0.9 good; 0.7–0.8 acceptable; 0.6–0.7 doubtful; and 0.5–0.6 poor [33]. We evaluated the inter-evaluator reproducibility for the scores obtained from each dimension and the total MTCCP score using the intraclass correlation coefficient (ICC). The ICC results were interpreted according to the Landis and Koch classification as follows: values of 0.81–1.00 indicated almost perfect agreement; values of 0.61–0.80 indicated considerable agreement; values of 0.42–0.60 indicated moderate agreement; values of 0.21–0.40 indicated fair agreement; values of 0.00–0.20 indicated low agreement; and values < 0 indicated poor agreement [34].

## Results

In terms of content validity, the CVI indexes were positive—higher than 0.8 (Table 1). With regard to construct validity, the KMO analysis yielded an index of 0.9 (*p* < 0.001), indicating the appropriateness of the data for PCA. Two factors with eigenvalues ≥1 were extracted by PCA and accounted for 80.69% of the overall variance. As shown in Table 2, the first factor (denoted clinical reasoning) accounted for 44.7% of the total variance and included 10 items with factor loadings ≥0.70. The second factor (professional behavior) accounted for 35.9% of the variance and included 10 items with factor loadings ≥0.5.

**Table 1:** Content validity index of the measurement tool for clinical competencies in physiotherapy

	Relevance	Sufficiency	Pertinence	Coherence	Clarity
	CVI	CVI	CVI	CVI	CVI
Professional behavior 1	1.00	0.90	1.00	1.00	0.74
Professional behavior 2	1.00	0.85	1.00	0.95	1.00
Professional behavior 3	0.90	0.90	0.84	0.90	0.85
Professional behavior 4	1.00	0.85	1.00	0.90	0.79
Professional behavior 5	1.00	0.95	1.00	0.95	0.90
Professional behavior 6	1.00	0.90	1.00	0.95	0.95
Professional behavior 7	1.00	1.00	1.00	1.00	1.00
Professional behavior 8	1.00	0.95	1.00	1.00	1.00
Professional behavior 9	0.95	0.85	0.95	0.95	0.69
Professional behavior 10	1.00	0.89	1.00	1.00	0.95
Clinical reasoning 1	1.00	1.00	1.00	1.00	1.00
Clinical reasoning 2	0.95	0.89	0.95	0.84	0.73
Clinical reasoning 3	0.94	0.83	0.94	0.89	0.94
Clinical reasoning 4	1.00	0.94	1.00	1.00	1.00
Clinical reasoning 5	0.95	0.95	0.95	0.95	1.00
Clinical reasoning 6	1.00	0.84	1.00	1.00	1.00
Clinical reasoning 7	1.00	1.00	1.00	0.95	0.95
Clinical reasoning 8	0.94	0.94	0.94	0.94	0.89
Clinical reasoning 9	0.94	0.94	0.94	1.00	0.88
Clinical reasoning 10	1.00	0.94	1.00	0.94	0.78

*CVI* Content validity index

**Table 2:** Factor pattern coefficients for principal component analysis with Varimax rotation and corrected item-total correlations on the 20 items of the MTCCP (*n* = 60)

	Item	F1	F2
Professional Behavior 1	Minimizes the actual risk of damage in itself and the population it serves.	0,67	**0,57**
Professional Behavior 2	Meets the ethical and bioethical principles of the professional practice.	0,58	**0,68**
Professional Behavior 3	Uses efficiently and adequately the physical and technological resources available in the practice setting.	0,59	**0,63**
Professional Behavior 4	Has assertive verbal, nonverbal, and written communication.	0,64	**0,52**
Professional Behavior 5	Establishes interdisciplinary academic relations for the benefit of his/her training process and of the user’s assistance.	0,53	**0,63**
Professional Behavior 6	Shows initiative and leadership in managing knowledge and organizing activities within the practice.	0,44	**0,78**
Professional Behavior 7	Shows continuous commitment to improvement for his/her personal and professional development.	0,35	**0,88**
Professional Behavior 8	Bases his/her professional undertaking on the best available scientific evidence.	0,50	**0,73**
Professional Behavior 9	Fully assumes the undertaken commitments typical of the professional performance and his/her role as a student.	0,50	**0,80**
Professional Behavior 10	Takes part meeting efficiency and quality in the administrative activities of his/her practice.	0,30	**0,85**
Clinical reasoning 1	Produces an initial hypothesis of the user’s clinical condition based on the available information: clinical records, observations, and interviews.	**0,81**	0,3
Clinical reasoning 2	Selects the tests and measures consistent with the user’s priorities and the best available scientific evidence.	**0,79**	0,47
Clinical reasoning 3	Applies the selected tests and measures skillfully.	**0,73**	0,43
Clinical reasoning 4	Analyzes the obtained information to produce a diagnosis of the user’s functional condition.	**0,78**	0,42
Clinical reasoning 5	Determines the physiotherapeutic prognosis that allows him/her to project goals and treatment plan.	**0,74**	0,49
Clinical reasoning 6	Establishes the general objective of the treatment plan according to the user’s diagnosis and prognosis.	**0,83**	0,40
Clinical reasoning 7	Structures the treatment plan taking the available resources and evidence into account.	**0,76**	0,51
Clinical reasoning 8	Applies the therapeutic strategies established in the treatment plan skillfully.	**0,76**	0,45
Clinical reasoning 9	Carries out educational strategies for body and movement in order to fulfill the set objectives.	**0,85**	0,32
Clinical reasoning 10	Evaluates the impact of his/her interventions and makes the required adjustments to the treatment based on the behavior of the relevant clinical variables.	**0,76**	0,546
	Eigenvalues	15,10	1,03
	% of variance	44,76	35,92

F1, Factor 1 (clinical reasoning) and F2, Factor 2 (professional behavior) and include the next sentence: Boldface numbers identify the relation between each item and its factor taking into account the factor loadings (F1 ≥ 0.70) (F2 - ≥0.5)

Based on 60 evaluations, 20 items of the MTCCP achieved internal congruence, with a Cronbach’s alpha coefficient of 0.982. The inter-evaluator reproducibility for clinical reasoning, professional behavior, and the total MTCCP score was almost perfect; the ICCs were 0.984, 0.930, and 0.983, respectively.

## Discussion

Assessing clinical competencies is important when preparing PT students for clinical practice [35–37]. A range of tools has been used to evaluate clinical competencies [38–41]. The literature on the assessment of the clinical performance of physiotherapy students in the South American context is limited. This is the first known study conducted in a Spanish-speaking country to develop a tool for assessing PT students in clinical practice and to measure its psychometric properties. In this study, the MTCCP has two categories: professional behavior and clinical reasoning. Our results showed that items 1 (minimizes the actual risk of damage itself and in the population served) and 4 (has assertive verbal, nonverbal, and written communication) may be considered factorially complex, because they showed similar loadings in both the professional behavior and clinical reasoning categories. Indeed, these components are strongly associated, as suggested by the significant and large correlation coefficient between these two factors. In the original validation studies [25], these items were assigned to the professional behaviors category. In our context, these items were also assigned to the professional behavior category. These results reinforce our concept of professional behavior: a set of attitudes and behaviors that reflect the physical therapist’s ethical commitment in providing health services.

Earlier studies have described assessment instruments for measuring the clinical performance of PT students in specific settings, such as Blue MACS [22], the CIET [25], or the CPI [20]. The CPI was found to be a valid and reliable instrument for assessing clinical competence in three areas: “Professional Practice,” “Patient Management,” and “Practice Management.” The MTCCP takes a different approach in that some items related to practice management are included in the professional behavior category according to our conceptual reference framework.

The MTCCP achieved internal congruence, with a Cronbach’s alpha coefficient of 0.982. The inter-evaluator reproducibility for clinical reasoning, professional behavior, and the total MTCCP score was almost perfect; the ICCs were 0.984, 0.930, and 0.983, respectively. These findings are in line with the CPI Cronbach’s alpha values of 0.99 and 0.97 [20, 36]. These results have important implications for PT clinical education. Educators must be committed to using valid assessment tools that measure their students’ performance in their clinical areas objectively, accurately, and consistently in terms of the prioritization of core clinical duties on a day-to-day basis [35].

The reliability level obtained by the MTCCP is greater than that reported for Blue MACS (0.78 and 0.83) [22] or for the CIET, which had an overall ICC value of 0.84 [37].

These values may be associated with the clarity of the evidence proposed for each item, which allows the evaluator to easily establish whether an evaluated student meets the criteria. Another reason for the reproducibility of the present results could be related to the discrete measurement scale of 1–5 used in this study. This scale allows a precise description of both the required level of supervision and the expected undergraduate achievement level. By contrast, the CPI [20] uses a visual analogue scale, and the Blue MACS [22] employs a Likert-type scale, which may be more subjective as it is based on the evaluator’s perception of agreement or disagreement.

The implication of these findings is that in clinical competence assessment in PT education, there is a high level of reliability in the assessment and scoring of undergraduate physiotherapy students’ performance in clinical placement when using a standardized assessment form with explicit guidelines.

This study is not without limitations. First, all of the students were selected from a single educational institution (Universidad del Rosario), and they volunteered to participate. This might have resulted in selection bias. In this study, we did not assess intra-evaluator reproducibility; to do so would have necessitated ensuring that the measurements were made in similar conditions in order to confirm their independence. However, it was not possible to carry out this assessment due to changes in the patients and in the students’ learning processes in the clinical setting. Nevertheless, future studies should make an effort to assess intra-evaluator reproducibility.

To further improve the validity and reliability of the instrument, we recommend investigating the scale in other institutional settings in order to ascertain whether its validity and integrity remain intact in different clinical settings. Based on these applications, a confirmatory factor analysis should be conducted to confirm that the items on the adjusted scales accurately reflect the underlying constructs.

In future research, the MTCCP instrument could be used both to help improve learning processes in individual PT students and to evaluate the effects of education on clinical performance. In the academic context, the MTCCP could help educators and students to identify which areas of learning students feel insecure with and therefore need further practice in. The MTCCP can be used in summative and formative evaluation processes: the summative evaluation would give an account of the student’s performance level based on the score obtained, whereas in the formative evaluation, without the pressure of a formal qualification, both the CI and the student identify the strengths and weakness of the student and make decisions that promote learning. The CI can establish the pedagogical strategies according to the student’s needs, and students appropriate these strategies in order to achieve significant learning in clinical competence training [42].

## Conclusions

The MTCCP is a valid and reliable instrument for assessing the performance of entry-level PT students in clinical areas in hospital settings. As such, it could be an important tool in PT education and research. In education, the MTCCP could be used to determine what skills the student feels less confident using and what additional training/learning opportunities could be provided. In research, it could be used to assess the impact of the curriculum and pedagogical strategies on students’ clinical performance. Further research is needed to determine whether the MTCCP has similar validity and reproducibility in other Spanish-speaking national and international PT programs.

## Additional file

Additional file 1: Appendix. Measurement Tool For Clinical Competencies In Physiotherapy (MTCCP). (DOCX 42 kb)

## Abbreviations

APTA: American PT Association
ASCOFI: Asociación Colombiana de Fisioterapia
CAPTE: Commission on Accreditation in PT Education
CPI: Clinical Performance Instrument
CVI: Content Validity Index
ICC: Intraclass Correlation Coefficient
KMO: Kaiser-Meyer-Olkin
MTCCP: Measurement Tool for Clinical Competencies in PT
WCPT: World Confederation for PT
