# Supplementary material for: Validity and reproducibility of a tool for assessing clinical competencies in physical therapy students
Source: BMC Med Educ. 2018 Nov 23;18:280. doi: 10.1186/s12909-018-1377-x (PMC6260878; doi:10.1186/s12909-018-1377-x)
Supplement: Supplementary file 1 — Appendix. Measurement Tool For Clinical Competencies In Physiotherapy (MTCCP). (DOCX 42 kb) [file 12909_2018_1377_MOESM1_ESM.docx]

**Appendix**

| **MEASUREMENT TOOL FOR CLINICAL COMPETENCIES IN PHYSIOTHERAPY (MTCCP)** | | | | |
| --- | --- | --- | --- | --- |
|  | **Setting:** | |  |  |
|  | **Student’s Name:** | | **Date:** |  |
|  | **Evaluated by:** | |  |  |
| **1. Dimension: Professional Behavior** | | | | |
| **#** | **Statement** | **Evidences** | | **Grade** |
| 1 | Minimizes the actual risk of damage in itself and the population it serves. | Uses the basic bio-security norms and implements the institution’s patient security policy; recognizes and minimizes the risks inherent to the test implementation, of the measurements and interventions taking care of the body mechanics. | |  |
| 2 | Meets the ethical and bioethical principles of the professional practice. | Acts responsibly; approaches with moral reasoning the ethical dilemmas; takes into account the needs and values of others; is accurate and consistent doing what he/she says he/she is going to do; manages information confidentially; and adheres to the ethical guidelines specific to physiotherapy (APTA and Law 528/1999) considering the user’s cultural context. | |  |
| 3 | Uses efficiently and adequately the physical and technological resources available in the practice setting. | Adequately uses software, hardware and elements intended to provide physiotherapy services (electrotherapy equipment, physical means, gymnasium); justifies the use of resources in accordance with the requirements of service, user and practice; knows the equipment technical specifications he/she uses; recognizes the proper functioning of the equipment he/she uses; and reports in due course equipment failures. | |  |
| 4 | Has an assertive verbal, nonverbal and written communication. | Communicates congruently and balanced expressing his/her convictions and defending his/her positions with argumentations; uses a posture that facilitates communications; and keeps eye contact with his/her interlocutor. | |  |
| 5 | Establishes interdisciplinary academic relations for the benefit of his/her training process and of the user’s assistance. | Interacts opportunely and respectfully with professionals in order to improve his/her professional performance and the clinical decision-making; participates in the working group considering his/her objective of study. | |  |
| 6 | Shows initiative and leadership in managing knowledge and organizing activities within the practice. | Proposes improvement actions; recognizes the own and the work group’s abilities within the context of the requirements of the practice setting; assertively solves situations that demand an immediate response. | |  |
| 7 | Shows continuous commitment to improvement for the personal and professional development. | Accepts, integrates and applies the received feedback given by the clinical instructor or the professionals in a constructive manner aiming at improving his/her performance; takes responsibly his/her learning and performs self-assessment of his/her own behavior. | |  |
| 8 | Bases his/her professional undertaking on the best available scientific evidence. | Is proficient in searching scientific literature to support his/her interventions and carries out a critical analysis of it in order to apply the knowledge produced by these studies to the assisted population. | |  |
| 9 | Fully assumes the undertaken commitments typical of the professional performance and his/her role as a student. | Composes documents and carries out activities under his/her charge all in a timely and a good quality basis, and in consistency with the education project of the academic program. | |  |
| 10 | Takes part meeting efficiency and quality in the administrative activities of his/her practice. | Keeps updated the documentation related to the conducted interventions (clinical record, indicators, invoicing and inventory) in accordance with the institutional guidelines of the specific practice setting and of the University. | |  |
| **TOTAL** | | Average of items multiplied by 0.4 factor | |  |
| **Scores: 5: Excellent**, does not require Clinical Instructor (**CI**) presence. **4**: **Good,** requires minimal CI presence. **3**: **Fair**, requires occasional CI presence. **2**: **Poor**, requires frequent CI presence. **1**: **Very poor**, requires permanent CI presence. | | | | |
|  |  |  | |  |

| **MEASUREMENT TOOL FOR CLINICAL COMPETENCIES IN PHYSIOTHERAPY (MTCCP)** | | | |
| --- | --- | --- | --- |
|  | **Setting:** |  |  |
|  | **Student’s Name:** |  | **Date:** |
|  | **Evaluated by:** |  |  |
| **2. Dimension: Clinical Reasoning** | | | |
| # | Statement | Evidences | Grade |
| 1 | Produces an initial hypothesis of the user clinical condition based on the available information: Clinical record, observation, and interview. | Integrates concepts of the basic and disciplinary sciences to select and understand in the expected time the available information referring to the user’s situation and his/her background. |  |
| 2 | Selects the tests and measures consistent with the user’s priorities and the best available scientific evidence. | Bases his/her test and measures selection on the psychometrical properties and the specificity of the tools, as well as the resource availability. |  |
| 3 | Applies skillfully the selected tests and measures. | Applies the tests and conducts the examinations with the proper technique: expected verbal commands, hand contact and positions (of user and physiotherapist) expected in each case. |  |
| 4 | Analyses the obtained information to produce a diagnosis of the user’s functional condition. | Argues his/her clinical judgement on the user’s condition in terms of deficiencies, activity limitations and participation constraints, based in the evaluation. |  |
| 5 | Determines the physiotherapeutic prognosis that allows him/her to project goals and treatment plan. | Argues the optimal recovery level that can be achieved and the required time to reach it based on the knowledge of the user’s conditions, the underlying pathology, the available evidence and the facilitators and barriers of the actual context. |  |
| 6 | Establishes the general objective of the treatment plan according to the user’s diagnosis and prognosis. | Establishes the goal of the specific functional reach for the user’s condition. |  |
| 7 | Structures the treatment plan taking the available resources and evidence into account. | Argues based on the physiological mechanisms of the treatment plan, defining the intervention intensity, frequency, volume, mode, method, and duration grounded on the user’s needs, preferences, and particularities, on the general objective, the evidence and the available resources. |  |
| 8 | Applies skillfully the therapeutic strategies established in the treatment plan. | Applies the intervention modalities with the proper technique: verbal commands, hand contact and positions (of user and physiotherapist) expected for each case. |  |
| 9 | Carries out education strategies for body and movement in order to fulfill the set objectives. | Argues the education strategies selection and implementation aimed at users and caregivers as part of the treatment plan. |  |
| 10 | Evaluates the impact of his/her interventions and makes the required adjustments to the treatment based on the behavior of the relevant clinical variables. | Argues the changes made to the treatment plan based on the findings of the reassessment of the established outcome measurements. |  |
| TOTAL | | Average of items multiplied by 0.6 factor |  |
| Scores: 5: Excellent, does not require Clinical Instructor (CI) presence. 4: Good, requires minimal CI presence. 3: Fair, requires occasional CI presence. 2: Poor, requires frequent CI presence. 1: Very poor, requires permanent CI presence. | | | |
